# Draft Genome Sequences of Four Aerobic Isobutane-Metabolizing Bacteria

**DOI:** 10.1128/MRA.01381-20

**Published:** 2021-05-06

**Authors:** Weijue Chen, Nicholas Faulkner, Christy Smith, Megan Fruchte, Michael Hyman

**Affiliations:** a Department of Plant and Microbial Biology, North Carolina State University, Raleigh, North Carolina, USA; University of Delaware

## Abstract

Here, we report the draft genome sequences of four aerobic gaseous alkane-oxidizing bacteria isolated from soil by enrichment culture using isobutane (2-methylpropane) as the sole carbon and energy source. The sequences all reveal microorganisms with multiple alkane-oxidizing monooxygenases, including soluble di-iron monooxygenases (SDIMOs), copper-containing monooxygenases (CuMMOs), and alkane hydroxylases (AHs).

## ANNOUNCEMENT

Bacteria that can oxidize gaseous nonmethane alkanes have been isolated from ethane (1), propane, (2) and *n*-butane (3, 4) enrichment cultures. In contrast, little is known about bacteria that grow on isobutane. This study reports the draft genome sequences of four isobutane-metabolizing bacteria.

Surface soil (≤10 cm below grade) was collected in sterile plastic tubes from 3 sites in North Carolina (GPS coordinates 35.787263, –78.674810; 35.789130, –78.683489; and 35.192664, –79.394702). Samples (5 g) from each site were incubated in sealed serum bottles (160 ml) containing mineral salts medium (MSM) (5) (25 ml) and isobutane (10% [vol/vol] in air). The cultures were incubated in the dark in an environmental shaker operated at 30°C and 150 rpm. After 14 days, samples (0.1 ml) of each enrichment were transferred to fresh MSM, and this cycle was repeated 3 times. Samples (0.1 ml) of each enrichment culture were then streaked onto MSM agar plates, which were then incubated for 14 days in dessicators containing isobutane (∼3% [vol/vol] in air). Each resulting colony type was then plated onto MSM agar plates, which were then incubated in dessicators containing isobutane (∼3% [vol/vol] in air). This process was repeated 3 times, and the purity of each isolate was determined by both Gram and acid-fast staining and microscopic observation. Out of a total of 18 isolates, 4 were subsequently selected for sequencing.

For DNA extraction, each isolate was grown in glass bottles (700 ml) sealed with screw caps and butyl rubber septa. The bottles contained MSM (100 ml) and isobutane (10% [vol/vol] in air). After 7 days, cells were harvested by centrifugation, and the sedimented cells were lysed in tubes (2 ml) containing silica beads (0.1 mm) using a FastPrep-24 bead beater (MP Biomedicals, California) operated at 4 m/s for 20 s. Total genomic DNA (gDNA) was extracted using a DNeasy UltraClean microbial kit (Qiagen, Maryland). The DNA was sheared using a g-TUBE (Covaris, Massachusetts) and size selected (5 kb) using the BluePippin size selection system (Sage Science, Inc., Massachusetts). Sequencing libraries were prepared using the PacBio Express template prep kit v2.0 (Pacific Biosciences, California). The libraries were sequenced using a PacBio Sequel single-molecule real-time (SMRT) cell, and raw reads were processed with the PacBio *de novo* assembly pipeline workflow on CLC Genomics Workbench v20.0.2. The genomes were annotated using the NCBI Prokaryotic Genome Annotation Pipeline (6), and genome completeness was assessed using BUSCO v4.1.2 and the corynebacteriales_odb10 database (7). Default parameters were used for all software tools, and the sequencing and assembly statistics, Benchmarking Universal Single-Copy Orthologs (BUSCO) analysis, and major genome characteristics of the four strains are summarized in Table 1.

**TABLE 1:** Genome information of 4 isobutane-metabolizing bacteria

Strain	Sequencing		Assembly		Characteristics						BUSCO analysis^*b*^		
	Total no. of reads	Total yield of reads (Mbp)	No. of contigs	*N*_50_ (bp)	Genome size (Mbp)	GC content (%)	Total no. of genes	Total no. of CDSs^*a*^	No. of rRNAs	No. oftRNAs	Complete and single copy (%)	Complete and duplicated (%)	Fragmented (%)
*Mycolicibacterium* sp. strain 2A	139,559	1,243	3	6,838,982	7.12	66.92	6,841	6,785	6	47	99.1	0.5	0.1
*Rhodococcus* sp. strain 3A	292,773	2,361	8	7,733,895	8.74	67.20	8,119	8,054	12	50	96.5	2.8	0.3
*Rhodococcus* sp. strain 4CI	189,816	1,737	6	4,181,179	6.59	70.20	6,011	5,941	13	54	98.0	1.2	0.4
*Rhodococcus* sp. strain 4CII	194,179	1,637	6	7,821,090	8.82	67.20	8,148	8,081	14	50	96.5	2.8	0.3

a CDSs, coding DNA sequences.

b BUSCO is a method for assessing the completeness of genome assemblies and annotations. It examines the presence of genes for numerous universal single-copy orthologs and designates them as either complete (within 2 standard deviations of expected size), duplicated (when the gene is found in more than one copy), or fragmented (for partially recovered genes).

**FIG 1:**
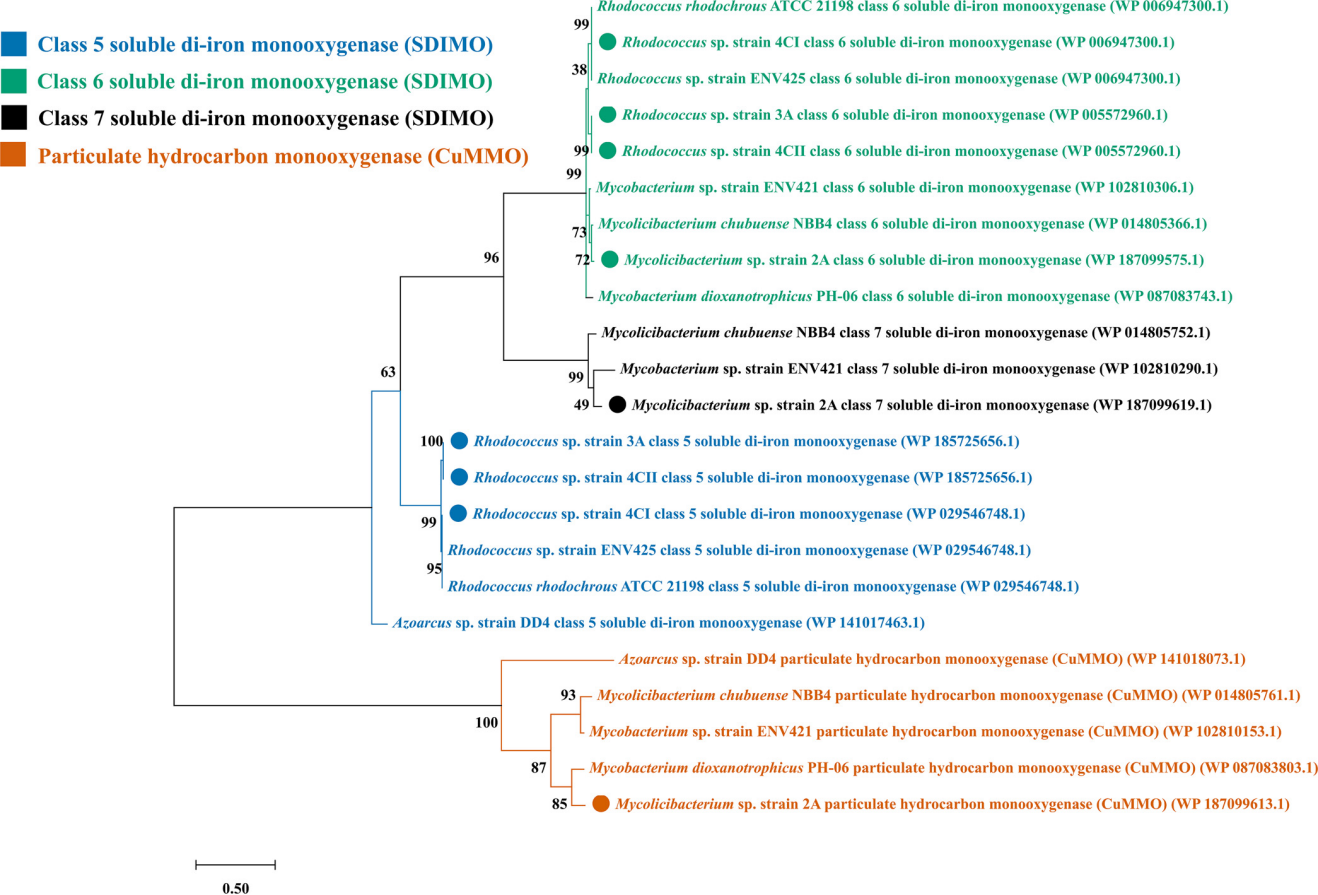
Phylogeny of monooxygenases in isobutane-metabolizing bacteria. Shown is a phylogenetic analysis of the putative gaseous alkane-oxidizing monooxygenases present in the genome sequences of the four bacterial strains sequenced in this study (*Mycolicibacterium* sp. strain 2A, *Rhodocococcus* sp. strain 3A, *Rhodocococcus* sp. strain 4CI, and *Rhodocococcus* sp. strain 4CII) and representative strains previously described in the literature. The analysis is based on the deduced amino acid sequences of the alpha subunits of the soluble di-iron monooxygenases (SDSIMOs) and A subunits of the copper-containing particulate hydrocarbon-oxidizing monooxygenases (CuMMOs). ClustalW and maximum likelihood analyses were conducted using MEGA X v10.1.8. The number at the nodes represents the bootstrap value by 1,000 bootstrap replicates. The accession numbers of each monooxygenase component in the NCBI RefSeq databases are provided in parentheses.

A BLAST comparison of 16S rRNAs from the genome sequences against the NCBI 16S rRNA database revealed that the isolates were all examples of frequently encountered gaseous alkane-oxidizing genera. All four genomes encoded complete operons for a group 6 soluble di-iron monooxygenase (SDIMO) (Fig. 1). Complete operons encoding other SDIMOs or copper-containing monooxygenases (CuMMOs) were also found in some but not all of the strains. Each genome also encoded one or more AlkB-like alkane hydroxylases (AH) (8).

### Data availability.

The complete genome sequences of the four isobutane-utilizing strains have been deposited in GenBank under the accession numbers CP059893 to CP059895 (*Mycolicibacterium* sp. strain 2A), JACJDF000000000 (*Rhodococcus* sp. strain 3A), JACCFD000000000 (*Rhodococcus* sp. strain 4CI), and JACCFE000000000 (*Rhodococcus* sp. strain 4CII). The GenBank assembly numbers for the genomes are GCF_014295435 (*Mycolicibacterium* sp. strain 2A), GCF_014230115 (*Rhodococcus* sp. strain 3A), GCA_014230025 (*Rhodococcus* sp. strain 4CI), and GCA_014256275 (4CII). The BioProject accession number for the genomes is PRJNA644947. The Sequence Read Archive (SRA) numbers are SRX9122159 (*Mycolicibacterium* sp. strain 2A), SRX9122160 (*Rhodococcus* sp. strain 3A), SRX9122161 (*Rhodococcus* sp. strain 4CI), and SRX9122162 (*Rhodococcus* sp. strain 4CII).
